# Mitral Regurgitation in Takotsubo Syndrome: A Comprehensive Narrative Review

**DOI:** 10.7759/cureus.96036

**Published:** 2025-11-03

**Authors:** Pranesh Gavali, Rushin S Parekh, Jahanvi G Kasodariya, Yamini Vala

**Affiliations:** 1 Internal Medicine, Seth Gordhandas Sunderdas Medical College, Mumbai, IND; 2 Internal Medicine, Gujarat Medical Education &amp; Research Society (GMERS) Medical College and Hospital, Sola, Ahmedabad, IND; 3 Internal Medicine, Surat Municipal Institute of Medical Education and Research, Surat, IND; 4 Internal Medicine, Government Medical College, Mahabubnagar, Mahabubnagar, IND

**Keywords:** cardiogenic shock, echocardiography, lv outflow tract obstruction, mitral regurgitation, stress cardiomyopathy, systolic anterior motion, takotsubo syndrome

## Abstract

Takotsubo syndrome (TTS) is an acute, transient, non-ischemic cardiomyopathy marked by circumferential wall‑motion abnormalities that typically extend beyond a single epicardial territory. Although most patients recover left ventricular function within weeks, the acute phase can be destabilized by mechanical complications and cardiogenic shock. Mitral regurgitation (MR) in TTS is frequent and clinically consequential, arising from systolic anterior motion (SAM) with dynamic left ventricular outflow tract obstruction (LVOTO) or from leaflet tethering driven by apical or mid-ventricular ballooning in the absence of obstruction. These mechanisms demand divergent strategies because inotropes and afterload reduction that may help pump failure can aggravate obstruction, whereas preload reduction that aids congestion can worsen gradients if LVOTO is present. This review synthesizes epidemiology, mechanisms, imaging pathways, hemodynamic phenotypes, management, special contexts, outcomes, and research priorities of MR in TTS using only the provided peer-reviewed sources. Transthoracic echocardiography anchors diagnosis by identifying SAM, quantifying MR, and measuring left ventricular outflow tract (LVOT) or intracavitary gradients, while transesophageal echocardiogram (TEE), cardiovascular magnetic resonance imaging (CMR), left ventriculography, and myocardial contrast echocardiography (MCE) provide complementary insights when acoustic windows are limited or when postoperative differentials must be adjudicated. Right ventricular involvement denotes a higher‑risk phenotype with more functional regurgitation and worse short-term outcomes, reinforcing the need for meticulous preload and afterload management. In patients with obstruction, therapy focuses on beta-blockers, careful volume expansion, and vasoconstrictors to restore stability. For patients with tethering-dominant MR, the priority is decongestion and customized afterload reduction. If shock persists despite these measures, temporary mechanical circulatory support can be used to bridge patients to recovery.

## Introduction and background

Takotsubo syndrome (TTS) commonly presents with chest pain or dyspnea and ECG changes that mimic acute coronary syndromes despite non-obstructive coronaries [1-3]. The phenotype includes classic apical ballooning and non-apical variants that emphasize heterogeneity and a circumferential distribution of dysfunction [1,2]. Emotional or physical stressors are frequent precipitants, and perioperative states represent potent triggers due to adrenergic surges and dynamic loading changes [1,3]. Acute complications include pulmonary edema, MR, left ventricular outflow tract obstruction (LVOTO), malignant arrhythmias, atrial fibrillation, right ventricular involvement, thromboembolism, and shock [1-4]. Among these, MR uniquely translates regional mechanics into elevated left atrial pressure and reduced forward output [4,5]. Mechanism classification by echocardiography is pivotal because therapeutic choices can have opposite effects in obstruction versus pump‑failure phenotypes [2-4].

## Review

Epidemiology and clinical burden

The prevalence of moderate-to-severe MR varies across cohorts and imaging timing, yet MR is repeatedly recognized as a frequent and clinically relevant accompaniment of the acute phase [1-3]. When present, MR correlates with pulmonary edema, hypotension, and escalation of respiratory and hemodynamic support [3-5]. Right ventricular involvement marks a higher‑risk phenotype with greater functional atrioventricular valve regurgitation and worse outcomes [6-9]. The burden of MR is most pronounced early when ventricular geometry is most distorted and adrenergic tone is highest, with attenuation as wall‑motion abnormalities resolve [2,3]. Even modest MR can be consequential in the presence of LVOTO because regurgitation and outflow gradients interact to reduce effective forward stroke volume [2,3,4]. In tethering‑dominant MR without obstruction, decongestion and afterload reduction can improve symptoms when blood pressure allows [5].

Incidence and outcomes

The prevalence of moderate to severe MR in TTS ranges from 15 to 25%, depending on the series (Table 1). MR has consistently been associated with Higher Killip class on admission, EF <30% at presentation with slower recovery, Increased use of intra-aortic balloon pump (IABP), and higher in-hospital mortality [10].

**Table 1:** Incidence of MR in Takotsubo Cardiomyopathy Studies [10]

N	Moderate–Severe MR (%)	Key Outcome
227	21.5%	Independent predictor of mortality
68	20%	Associated with SAM, low EF

Pathophysiology of MR in Takotsubo Syndrome (TTS)

The pathophysiology of MR in TTS stems from the hallmark regional wall motion abnormalities: apical akinesis or ballooning contrasted with basal hypercontractility. This dyssynchrony disrupts mitral valve apparatus integrity, leading to two predominant mechanisms of acute MR.

*Mechanistic Framework: Systolic Anterior Motion-Left Ventricular Outflow Tract Obstruction* (*SAM‑LVOTO)*

SAM-mediated MR arises when hyperdynamic basal contraction and a narrowed outflow tract create a Venturi effect that displaces the anterior leaflet toward the septum [2,3]. Leaflet‑septal contact produces late‑systolic peaking gradients and a posteriorly directed eccentric jet that intensifies with adrenergic stimulation and hypovolemia [2,3].

**Figure 1:**
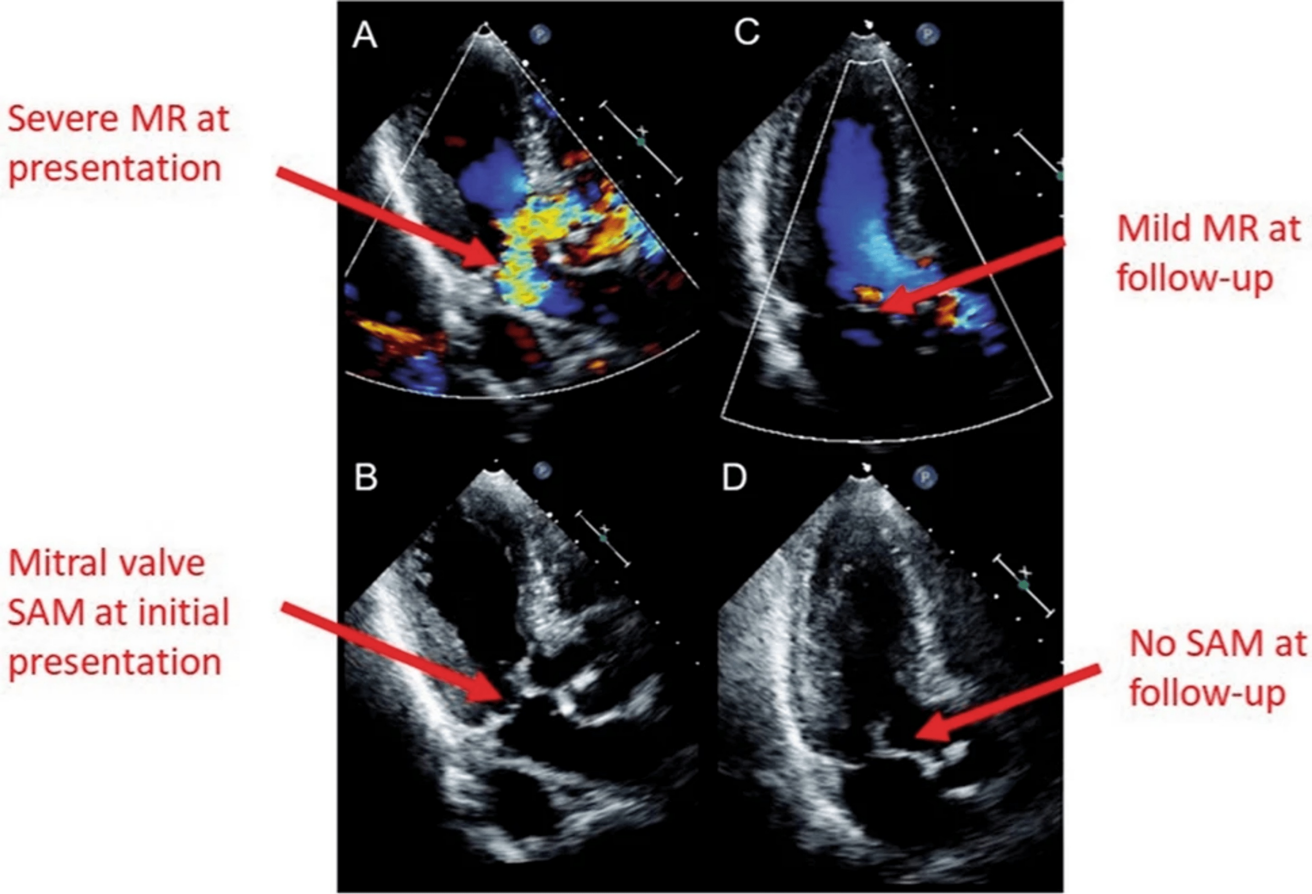
Two-dimensional transthoracic echocardiography, apical long-axis views, in patients with mitral regurgitation (MR) due to systolic anterior motion (SAM) of the mitral valve. (A) Severe MR at initial presentation. (B) SAM of the mitral valve at initial presentation. (C) Only mild MR was found at follow-up. (D) SAM of the mitral valve was not found at follow-up [10].

Afterload reduction and positive inotropy augment gradients and MR by increasing flow velocities and contractile forces [2,3,4]. Mechanism‑appropriate therapy emphasizes beta‑blockade, cautious volume expansion, and vasoconstriction to enlarge the cavity and dampen SAM [2,3,4]. Diuresis must be judicious because excessive preload reduction can shrink cavity size and worsen obstruction and regurgitation [2,3]. Mitral interventions are rarely required because MR typically regresses as obstruction resolves (Figure 1) [2,5].

Mechanistic Framework: Tethering‑Dominant MR

**Figure 2:**
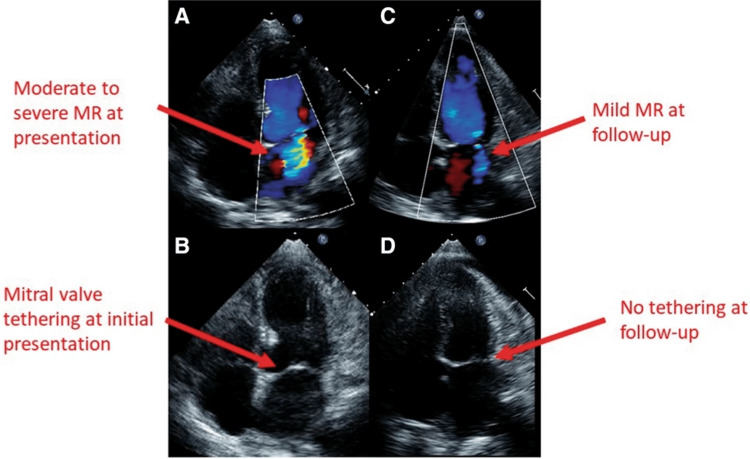
Two-dimensional transthoracic echocardiography, apical four-chamber views, in patients with functional mitral regurgitation (MR). (A) Moderate to severe MR at initial presentation. (B) Mitral valve tethering at initial presentation. (C) Only mild MR was found at follow-up. (D) Mitral valve tethering was not found at follow-up [10].

Tethering‑dominant MR occurs when apical or mid-ventricular ballooning displaces papillary muscles and lengthens chordae, restricting leaflet coaptation without significant obstruction [2,5]. This geometry often yields a central jet on color Doppler and is most severe when tenting height and leaflet restraint are maximal early after symptom onset [2,5]. Afterload reduction can decrease regurgitant fraction when systemic pressure permits, while decongestion mitigates left atrial hypertension [5]. Because LVOTO is absent, carefully selected inodilators can support forward flow in pump failure if blood pressure is tolerated [3,4,5]. Serial echocardiography documents rapid improvement as wall‑motion abnormalities regress and geometry normalizes (Figure 2) [2,3].

Mixed Phenotypes and Perioperative Dynamics

Mixed phenotypes with both tethering and SAM occur in small cavities with basal septal prominence or latent predisposition to dynamic obstruction [2,3,8]. Real-time Doppler interrogation and meticulous titration of preload and afterload are required to avoid iatrogenic worsening of either component [2,3,4]. Perioperative TTS highlights these swings because catecholamines and fluid shifts can toggle patients between obstruction-dominant and pump-failure states with evolving MR severity [3,9,10].

Imaging pathways and quantification

TTE is first‑line for mechanism identification and MR quantification using an integrated approach with color jet analysis, vena contracta, PISA when feasible, pulmonary vein flow, and supportive Doppler signatures [2,5]. Continuous‑wave Doppler across the LVOT measures dynamic gradients, while pulsed‑wave mapping can localize mid‑cavity acceleration in variant patterns [2,3]. Strain imaging may reveal circumferential impairment that transcends a single vascular territory, supporting TTS over infarction [2]. Transesophageal echocardiogram (TEE) clarifies SAM trajectory, commissural anatomy, and leaflet coaptation in intubated or postoperative patients when TTE windows are limited [2,3,9,10,11]. Left ventriculography documents ballooning morphology and permits simultaneous hemodynamic pullbacks to detect obstructive physiology [2,12]. Cardiovascular magnetic resonance imaging (CMR) refines morphology and tissue characterization and helps exclude myocarditis or infarction while demonstrating reversible myocardial edema typical of TTS [2]. Myocardial contrast echocardiography (MCE) can show preserved microvascular perfusion in akinetic segments, informative in postoperative differentials [11]. Serial reassessment is essential because MR severity and LVOT gradients can evolve quickly as adrenergic tone and loading conditions change [2,3].

Mechanism-targeted management

In obstruction, priorities are to blunt hypercontractility, maintain or slightly increase afterload, and avoid further cavity shrinkage [2,3,4]. Short-acting beta‑blockers reduce SAM contact time and gradients, improving MR and forward output when blood pressure allows [2,3]. Phenylephrine may be useful for hypotension because it augments afterload without increasing inotropy [3,4]. Dobutamine and dopamine can worsen gradients and MR and should be avoided unless no obstruction is present and the benefits clearly outweigh the risks [2,3,4]. In tethering‑dominant MR without obstruction, decongestion and afterload reduction reduce regurgitant volume when pressure permits [5]. If pump failure persists with adequate pressure, selected inodilators can be considered while anticipating rapid improvement as geometry normalizes [3,4,5]. Refractory shock may require temporary mechanical circulatory support with devices that do not exacerbate obstruction, serving as a bridge to recovery [3,4,9,10].

Special contexts and overlapping conditions

Perioperative TTS after mitral surgery illustrates labile hemodynamics where catecholamines, fluid shifts, and ventilation changes can swing patients between obstruction and pump failure [9,10,11]. TEE and MCE are decisive for differentiating TTS from perioperative infarction and for aligning therapy with the mechanism [10,11]. Coexistent hypertrophic cardiomyopathy predisposes to exaggerated SAM‑LVOTO during TTS and requires meticulous avoidance of inotropes and careful volume optimization [3,8]. Right ventricular involvement portends higher morbidity and more frequent functional regurgitation and mandates fine-tuned preload and afterload management [6]. Extensive apical akinesis carries a risk of mural thrombus and systemic embolization, warranting anticoagulation when thrombus is identified or the risk is high [7].

Outcomes and follow-up

Most MR in TTS resolves in parallel with recovery of wall‑motion abnormalities and normalization of ventricular geometry over days to weeks [2,3,9,10]. Nevertheless, significant MR at presentation is a marker of hemodynamic vulnerability and is associated with a greater risk of pulmonary edema, shock, and intensive care utilization [3,4,5]. Follow-up imaging confirms the trajectory and detects mural thrombus in extensive apical akinesis, which warrants anticoagulation consideration [2,5,7]. Residual MR after recovery should prompt reassessment for structural disease or persistent adverse geometry [2,5].

Practical bedside algorithm

Confirm or strongly suspect TTS based on presentation, ECG, biomarkers, and imaging that demonstrates non-coronary distribution wall‑motion abnormalities [1,2,3]. Identify and grade MR using an integrated echocardiographic approach and determine the dominant mechanism by searching for SAM and measuring LVOT or intracavitary gradients [2,3,5]. Treat obstruction with beta‑blockade, cautious fluids, and vasoconstriction while avoiding pure inotropes and excessive diuresis [2,3,4]. Treat tethering‑dominant MR without obstruction with decongestion and afterload reduction tailored to blood pressure, and consider noncatecholaminergic support for pump failure [2,3,5]. Reassess frequently, escalate to mechanical support when shock persists, and consider anticoagulation for thrombus or high-risk patterns [2,3,4,7,9,10].

Knowledge gaps and future directions

Standardization of MR quantification under dynamic conditions and validation of mechanism-specific imaging markers are needed [2,3]. Mechanism-oriented trials comparing pharmacologic sequences and device strategies in obstruction versus tethering‑dominant MR could refine care [3,4,5]. The role of edge-to-edge repair in refractory acute MR from TTS warrants cautious study in clearly defined phenotypes [5]. Registries integrating imaging, hemodynamics, and outcomes, including right ventricular involvement, will enable precision management and improve prognostication [1,2,4,6].

Practice points for the acute phase

Bedside echo‑guided titration of preload, afterload, and beta‑blockade aligns therapy with the dominant mechanism and avoids iatrogenic deterioration [2,3,4,13]. Clear documentation that links mechanism, MR grade, gradients, and hemodynamic responses supports safe handoffs and consistent decisions across teams [2,3,13]. Frequent reassessment is essential during early hospitalization because MR severity, gradients, and blood pressure can shift within hours [2,3,4,14]. Invasive mitral procedures are rarely indicated because MR is typically reversible once geometry and obstruction improve [2,5,15]. Right ventricular involvement requires careful avoidance of excessive afterload and maintenance of adequate coronary perfusion pressure [6,16]. Perioperative cases benefit from early TEE to define the mechanism and to avoid inotrope-driven worsening of dynamic obstruction [3,9,10,11,17]. MCE can demonstrate preserved microvascular perfusion in akinetic segments and reassure against infarction when angiography is deferred [11,18]. Anticoagulation should be individualized when mural thrombus is present or the risk is high in extensive apical akinesis [7,19]. Recovery expectations should be communicated to patients and teams, emphasizing the transient nature of TTS and the usual regression of MR with LV recovery [1,2,3]. Structured follow-up with imaging confirms resolution and identifies outliers who may harbor alternative structural mitral disease [2,5,20].

## Conclusions

Mitral regurgitation in TTS is more than a transient bystander. It often determines how unstable or safe a patient’s journey through the acute phase will be. What makes it particularly challenging is that the same clinical picture can arise from very different mechanisms: SAM with obstruction, or tethering from distorted ventricular geometry. Each calls for opposing therapies, which means that guessing is dangerous and imaging is indispensable. At the bedside, the art lies in watching the heart closely using echocardiography not once, but repeatedly, because MR severity can shift within hours as adrenergic tone and loading conditions change. Small adjustments in fluids, afterload, or beta-blockade can make the difference between deterioration and recovery. Most patients improve dramatically as wall-motion abnormalities resolve, yet the presence of MR at presentation should always alert clinicians to heightened risk.

Looking ahead, standardizing MR assessment in dynamic states, validating mechanism-specific markers, and testing tailored therapies in clinical studies will move the field from empiricism to precision. Until then, a careful, mechanism-guided approach anchored in imaging, reassessment, and restraint remains the surest path to guide patients safely through the storm of TTS toward recovery.
